# Relationship between serum uric acid and internal carotid resistive index in hypertensive women: a cross-sectional study

**DOI:** 10.1186/1471-2261-12-52

**Published:** 2012-07-16

**Authors:** José Alexandre Addeo Cipolli, Maria Carolina Ferreira-Sae, Rafael Prado Martins, José Alexandre Pio-Magalhães, Vera Regina Bellinazzi, José Roberto Matos-Souza, Wilson Nadruz Junior

**Affiliations:** 1 Department of Internal Medicine, School of Medical Sciences, State University of Campinas, São Paulo, Brazil; 2 Departamento de Clínica Médica, Faculdade de Ciências Médicas, Universidade Estadual de Campinas, Cidade Universitária “Zeferino Vaz”, 13081-970, Campinas, SP, Brasil

**Keywords:** Gender, Uric acid, Carotid artery, Resistive index, Intima-media thickness

## Abstract

**Background:**

The impact of serum uric acid (SUA) on arteries of hypertensive subjects remains to be fully established. This study investigated the relationship between SUA and carotid structural and hemodynamic parameters in hypertensive men and women.

**Methods:**

Three hundred and thirty eight patients (207 women and 131 men) were cross-sectionally evaluated by clinical, laboratory, hemodynamic and carotid ultrasound analysis. Common carotid diameters, circumferential wall tensions, Young’s Elastic Modulus, Stiffness Index, Arterial Compliance and intima-media thickness (IMT) were determined. Internal carotid artery resistive index (ICRI), a hemodynamic measure that reflects local vascular impedance and microangiopathy, was also assessed.

**Results:**

Univariate analysis showed no significant correlation of SUA with carotid diameters, elasticity/stiffness indexes, IMT and circumferential wall tensions in both genders. Conversely, SUA correlated with ICRI (r = 0.34; *p* < 0.001) in women, but not in men, and hyperuricemic women presented higher ICRI than normouricemic ones (0.684 ± 0.007 vs. 0.649 ± 0.004; *p* < 0.001). Stepwise and logistic regression analyses adjusted for potential confounding factors showed that ICRI was independently associated with SUA and hyperuricemia in women.

**Conclusions:**

This study demonstrated that SUA was associated with ICRI in hypertensive women, suggesting that there might gender-related differences in the relationship between SUA and vascular damage in subjects with systemic hypertension.

## Background

Serum uric acid (SUA) has been considered an independent predictor of cardiovascular events in hypertensive patients [1]. Experimental data also showed that uric acid stimulates proliferation, inflammation and oxidative stress in vascular smooth-muscle cells, induces endothelial dysfunction and activates the renin–angiotensin system [1,2]. These observations have supported the notion that SUA might be not only a marker but also a cardiovascular risk factor [1,3]. Nevertheless, the impact of SUA on the structure of large arteries of hypertensive subjects remains controversial, since this variable has been associated with subclinical atherosclerosis and aortic stiffness in some hypertensive populations [4-6], but not in others [7,8]. In this context, it can be speculated that alternative mechanisms rather than remodeling of large arteries could contribute to explain the higher cardiovascular risk attributed to SUA in hypertensive subjects. The resistive index of an artery is a hemodynamic measure considered to reflect its vascular impedance [9], and higher resistive index values consist in a manifestation of local arteriolopathy [9,10]. Recent data demonstrated an independent relationship between SUA and renal artery resistive index in hypertensive subjects [10,11], indicating that SUA might be associated with microvascular damage and/or dysfunction in clinical settings. However, the impact of SUA on the resistive index of other arteries remains unknown.

In general, there has been no difference in the association between SUA and cardiovascular risk in men and women [1,2]. However, some reports suggested that there might be gender-related differences in uric acid-related adverse cardiovascular prognosis. Data from the LIFE trial demonstrated that the association between the level of SUA and cardiovascular outcomes was significant only in women after adjustment for the Framingham risk score [12]. In addition, SUA was found to be independently associated with silent brain infarcts in women, but not in men [13].

### Aim

The present study investigated the relationship between SUA and carotid hemodynamic and structural parameters in hypertensive subjects and the role of gender in this regard.

## Methods

### Study subjects

Three hundred and thirty eight hypertensive subjects (207 women and 131 men) were cross-sectionally evaluated by clinical, laboratory, hemodynamic and carotid ultrasound analysis. The subjects were randomly selected from those attending an outpatient clinic of a university hospital. Exclusion criteria were age under 18 years, neoplastic disease and identifiable causes of secondary hypertension. The research was carried out in accordance with the Declaration of Helsinki of the World Medical Association. This study was approved by the Human Research Ethics Committee of the State University of Campinas. All subjects gave written informed consent to participate.

### Clinical and laboratory data

Body mass index was calculated as body weight divided by height squared (kg/m^2^). Waist circumference was measured at the midpoint between the lowest rib and the iliac crest. Fasting blood total cholesterol, low-density-lipoprotein cholesterol, high-density-lipoprotein cholesterol, triglycerides, creatinine, glucose and C-reactive protein levels were measured using standard laboratory techniques [14]. Creatinine clearance was estimated by the Cockroft-Gault formula. SUA analysis was performed by an enzymatic assay (Roche Diagnostics, Mannheim, Germany). Definition of hyperuricemia was a SUA level >7.0 mg/dL for men and >5.7 mg/dL for women [15].

Hypertension was defined as systolic blood pressure ≥140 mmHg or diastolic blood pressure ≥90 mmHg or current antihypertensive medication use. Diabetes mellitus was diagnosed if fasting blood glucose was ≥126 mg/dL or when participants were taking hypoglycemic medications, whereas metabolic syndrome was defined in accordance with the National Cholesterol Education Program Panel III criteria [16]. Women with reported amenorrhea for more than 12 months, except for pregnancy, were identified as postmenopausal.

### Systemic hemodynamic data

Blood pressure and heart rate were measured using a validated digital oscillometric device (HEM-705CP; Omron Healthcare, Japan). Systolic volume was generated from Doppler interrogation of transaortic flow at the aortic annular level and aortic cross-sectional area using a Vivid 3 Pro apparatus (General Electric, USA) equipped with a 2.5-MHz transducer [17]. Cardiac output was calculated as systolic volume x cardiac frequency, while peripheral vascular resistance was obtained by the formula: mean blood pressure/cardiac output.

### Carotid analysis

Carotid ultrasonography was performed by a single physician using a Vivid 3 Pro apparatus equipped with a 10-MHz linear-array transducer as previously described [18]. A region 2 cm proximal to the carotid bifurcation was identified, and the intima-media thickness (IMT) of the far wall was evaluated as the distance between the lumen–intima interface and the media–adventitia interface. All measurements were made using an automatic border recognizer (Vivid 3 Pro IMT software analyzer) on still images obtained during the sonographic scanning. End-diastolic and peak-systolic internal common carotid artery diameters were obtained by continuous tracing of 3 cycles and averaged. The ICRI was calculated as follows: 1–[minimum diastolic velocity/maximum systolic velocity] [9]. Common carotid artery IMT and diameters as well ICRI were obtained as the average from both right and left arteries measurements. Carotid ultrasound and concomitant brachial blood pressure measurements were used to calculate Young’s Elastic Modulus, Artery Compliance and Stiffness Index [19] and peak and mean circumferential wall tension [20].

Young’s Elastic Modulus gives an estimate of arterial stiffness that is independent of wall (intima-media) thickness by the formula: ([systolic blood pressure – diastolic blood pressure] x diastolic diameter)/([systolic diameter – diastolic diameter]/IMT). Artery Compliance measures the ability of the arteries to expand as a response to pulse pressure caused by cardiac contraction and relaxation and was calculated as: ([systolic diameter – diastolic diameter]/diastolic diameter)/(systolic blood pressure – diastolic blood pressure). Stiffness Index is considered to be relatively independent of blood pressure and was calculated by the formula: (systolic blood pressure/diastolic blood pressure)/([systolic diameter – diastolic diameter]/diastolic diameter) [19].

Circumferential wall tension measurements were calculated according to Laplace’s law: Peak circumferential wall tension = systolic blood pressure x peak-systolic internal diameter/2; Mean circumferential wall tension = mean blood pressure x ((systolic internal diameter + 2 x diastolic internal diameter)/3)/2.

Intraobserver and interobserver carotid IMT and diameters variabilities were <5%, while intraobserver and interobserver variabilities of ICRI measurements were <4%.

### Statistical analysis

Continuous variables with and without normal distribution are presented as mean ± standard error and median (25–75th percentile), respectively. The Kolmogorov–Smirnov test was used to test for normal distribution of the variables. *χ*2 was used to compare categorical variables whereas unpaired *t*-test and Mann–Whitney test compared continuous variables with and without normal distribution, respectively. Assessment of univariate correlations between variables was examined using Pearson’s correlation coefficient for normally distributed data and Spearman’s rank correlation coefficient for non-normally distributed data. Fisher’s z-test was used to compare correlation coefficients. Forward stepwise and logistic regression analysis evaluated the independent predictors of ICRI. Variables that exhibited significant correlation coefficients (*p* < 0.05) with ICRI were included as independent variables in stepwise regression analysis, while variables that were statistically different between hyperuricemic and normouricemic women were included as independent variables in logistic regression analysis. A *p-*value <0.05 was considered significant.

## Results and Discussion

Clinical, hemodynamic and laboratory characteristics of enrolled subjects are presented in Table 1, while carotid features are shown in Table 2. Results of univariate analysis between SUA and carotid variables are presented in Table 3. SUA exhibited no significant correlation with common carotid artery diameters, Young’s Elastic Modulus, Artery Compliance and Stiffness Index, IMT and circumferential wall tension measurements in both genders. Conversely, SUA correlated with ICRI (r = 0.34; *p* < 0.001) in females but not in males (r = −0.11; *p* = 0.19). The difference between these aforementioned correlation coefficients was statistically significant (*p* < 0.001) as assessed by Fisher’s z-test (Table 3). Further univariate analysis demonstrated that ICRI also correlated with pulse pressure (r = 0.44; *p* < 0.001), age (r = 0.38; *p* < 0.001), menopause status (r = 0.29; *p* < 0.001), creatinine clearance (r = −0.27; *p* < 0.001), creatinine (r = 0.23; p = 0.001), C-reactive protein (r = 0.22; *p* = 0.003), body mass index (r = 0.19; *p* = 0.007) and smoking (r = 0.15; *p* = 0.03) in women. SUA and ICRI showed no significant relationship with systemic vascular resistance. Similarly, results of Spearman’s correlation analysis revealed that carotid variables did not correlate with use of any antihypertensive medication, statins or allopurinol in both genders.

**Table 1:** Clinical, hemodynamic and laboratory features of hypertensive patients

**Variable**	**Women (n = 207)**	**Men (n = 131)**
Age, years	56.7 ± 0.9	59.1 ± 1.0
Body mass index, kg/m^2^	31.5 ± 0.4	29.8 ± 0.5^†^
Waist, cm	99.0 ± 1.0	102.3 ± 1.1*
Systolic blood pressure, mmHg	143.6 ± 1.7	140.8 ± 2.1
Diastolic blood pressure, mmHg	79.5 ± 1.2	76.6 ± 1.5
Pulse pressure, mmHg	64.1 ± 1.3	64.9 ± 1.6
Cardiac output, L/min	5.26 ± 0.09	5.73 ± 0.03^†^
Systemic vascular resistance, dynes·sec·cm^-5^	1604 ± 30	1427 ± 34^‡^
Metabolic syndrome, %	69	65
Diabetes mellitus, %	29	27
Smoking, %	6	14*
Menopause, %	75	____
Previous stroke, %	7	11
Serum uric acid, mg/dL	5.4 ± 0.1	6.7 ± 0.2^‡^
Creatinine, mg/dL	0.79 ± 0.02	1.12 ± 0.03^†^
Creatinine clearance, mL/min	99.0 ± 2.4	87.9 ± 3.0^†^
Low-density lipoprotein cholesterol, mg/dL	114.1 ± 2.5	103.5 ± 3.1^†^
High-density lipoprotein cholesterol, mg/dL	53.9 ± 1.2	46.8 ± 1.2^‡^
Triglycerides, mg/dL	128 (92)	129 (101)
Fasting blood glucose, mg/dL	96 (29)	100 (28)
C-reactive protein, mg/dL	0.39 (0.66)	0.44 (0.40)*
Allopurinol, %	5	6
Diuretics, %	76	84
Beta-blockers, %	47	48
Calcium-channel-blockers, %	52	48
ACEI or ARB, %	80	85
Statins, %	20	24

**Legend:** ACEI or ARB – angiotensin-converting-enzyme inhibitors or angiotensin-receptor blockers; *p < 0.05; ^†^*p* < 0.01 and ^‡^*p* < 0.001 compared to women.

**Table 2:** Carotid features of hypertensive patients

**Variable**	**Women (n = 207)**	**Men (n = 131)**
Common carotid features		
Intima-media thickness, mm	0.75 ± 0.01	0.79 ± 0.01*
Diastolic diameter, mm	5.4 ± 0.1	5.9 ± 0.1^‡^
Systolic diameter, mm	6.1 ± 0.1	6.7 ± 0.1^‡^
Young’s Elastic Modulus, mmHg·mm	238 ± 8	270 ± 12*
Artery Compliance, %/10 mmHg	2.21 ± 0.07	2.28 ± 0.13
Stiffness Index	5.16 ± 0.18	4.97 ± 0.18
Peak CWT, 10^4^ dynes/cm	5.9 ± 0.1	6.6 ± 0.1^‡^
Mean CWT, 10^4^ dynes/cm	3.8 ± 0.1	4.3 ± 0.1^‡^
Internal carotid resistive index	0.662 ± 0.004	0.673 ± 0.005

**Legend:** CWT – circumferential wall tension. *p < 0.05 and ^‡^*p* < 0.001 compared to women.

**Table 3:** Univariate analysis between serum uric acid and common carotid artery features

**Variable**	**Female (n = 207)**	**Male (n = 123)**	***p*(Z-test)**
Intima-media thickness	0.10	0.01	0.42
Diastolic diameter	0.14	0.08	0.59
Systolic diameter	0.13	0.04	0.42
Young’s Elastic Modulus	0.14	−0.03	0.13
Artery Compliance	−0.02	0.02	0.72
Stiffness Index	0.05	−0.06	0.33
Peak circumferential wall tension	0.09	−0.02	0.33
Mean circumferential wall tension	0.03	0.10	0.53
Resistive index (Internal carotid artery)	0.34*	−0.11	<0.001

**Legend:** * p < 0.001. Z-test was used to compare correlation coefficients between genders.

Clinical and carotid features of enrolled subjects were then evaluated according to the presence or not of hyperuricemia. No significant differences in carotid variables were detected between normouricemic and hyperuricemic men. On the other hand, hyperuricemic women presented similar carotid and clinical features in comparison to normouricemic ones, except for higher ICRI, body mass index, age, creatinine and triglycerides values and higher prevalence of metabolic syndrome and menopause (Table 4).

**Table 4:** Clinical and carotid features of hypertensive women according to the presence or not of hyperuricemia

**Variable**	**Normouricemic** **(n = 131)**	**Hyperuricemic** **(n = 76)**	***P***
Serum uric acid, mg/dL	4.5 ± 0.1	6.9 ± 0.2	<0.001
Internal carotid resistive index	0.649 ± 0.004	0.684 ± 0.007	<0.001
Body mass index, kg/m^2^	30.3 ± 0.5	33.6 ± 0.7	<0.001
Creatinine, mg/dL	0.76 ± 0.01	0.86 ± 0.03	<0.001
Metabolic syndrome, %	62	82	0.004
Triglycerides, mg/dL	131.5 ± 5.9	157.8 ± 8.7	0.009
Age, years	55.2 ± 1.2	59.9 ± 1.4	0.01
Menopause, %	68	82	0.02
Waist circumference, cm	97.5 ± 1.3	102.1 ± 1.4	0.02

**Legend.** No other studied clinical or carotid variable was statistically different between normouricemic and hyperuricemic women.

Stepwise regression analysis was performed to evaluate whether SUA was independently related to ICRI in hypertensive women. A significant association between ICRI and SUA was found in a model that also included pulse pressure, age, C-reactive protein, body mass index, smoking, creatinine clearance and menopause as independent variables (Table 5). Forced inclusion of antihypertensive classes, allopurinol and systemic vascular resistance in this model did not change the results. Likewise, logistic regression analysis demonstrated that ICRI ≥ 0.66 was independently associated with hyperuricemia [Exp(B) (95% C.I.) = 3.35 (1.62–6.53); *p* < 0.001] in a model that still included body mass index ≥ 30 kg/m^2^, age > 55 years, triglycerides > 150 mg/dL, creatinine clearance > 60 mL/min, menopause and metabolic syndrome as independent variables. On the other hand, forced inclusion of antihypertensive classes and allopurinol in the logistic regression model did not change the results.

**Table 5:** Stepwise regression analysis for internal carotid resistive index in hypertensive women

**Variable**	***R***^***2***^**change**	***P***
Pulse pressure	0.221	<0.001
Serum uric acid	0.079	<0.001
Age	0.042	<0.001
C-reactive protein	0.019	0.02

**Legend.** The model also included body mass index, creatinine clearance, smoking and menopause as independent variables. Creatinine was not included in the regression model because of multicollinearity with creatinine clearance. Further inclusion of antihypertensive classes, allopurinol and systemic vascular resistance as independent variables did not change the results.

Several lines of evidence demonstrated that SUA is an independent predictor of worse cardiovascular outcomes in hypertensive patients [1,3]. In addition, clinical studies have indicated that SUA might be a risk factor for cardiovascular diseases. In this regard, treatment with allopurinol not only lowered SUA levels but also reduced blood pressure levels of adolescents with newly diagnosed essential hypertension [21], improved endothelial dysfunction in subjects with chronic heart failure [22] and slowed the progression of renal disease in patients with chronic kidney disease [23]. In the present report, we investigated the relationship between SUA and several carotid hemodynamic and structural parameters in a sample of hypertensive patients. Noticeably, SUA exhibited an independent association with ICRI solely in hypertensive women, but no relationship with carotid structural parameters and circumferential wall tension in both genders. In addition, hyperuricemic subjects presented similar carotid parameters in comparison to normouricemic ones, except for higher ICRI values in women. ICRI is a hemodynamic measure thought to reflect intracranial vascular impedance [9,24] and is a predictor of cardiovascular mortality and morbidity, at least comparable to the well-established IMT [9,25]. It has been assumed that increases in ICRI occur before the thickening of the intima-media complex, consisting in a manifestation of arteriolopathy in the territory irrigated by the artery [9,10]. Thus, it can be speculated that SUA was associated with intracranial microvascular damage and/or dysfunction in hypertensive women. In agreement with this assumption, data from a recent report demonstrated that in a sample of women with high prevalence of hypertension, SUA was related to silent brain infarction [13], which may be considered a manifestation of cerebral microangiopathy.

The relationship between SUA and ICRI can be justified by the ability of uric acid to induce vasoconstriction and vascular remodeling. Uric acid is known to induce proliferation and oxidative stress in cultured vascular smooth-muscle cells, promote endothelial dysfunction and activate the renin–angiotensin system [1,2]. Conversely, our data from stepwise regression analysis revealed that ICRI was not only related to SUA but also to C-reactive protein levels in hypertensive women, indicating that inflammatory mechanisms played a role in microvascular remodeling. Interestingly, results of univariate analysis also revealed a significant correlation between SUA and C-reactive protein levels in hypertensive women (r = 0.27; *p* < 0.001), a finding that not only strengthens the idea that SUA is associated with subclinical systemic inflammation in hypertensive subjects [8], but also suggests that such association may contribute to explain the relationship between SUA and ICRI in our sample. In addition, the association between SUA and ICRI was found to be independent of systemic vascular resistance, indicating that selective arterial territories may be sensitive to SUA.

It was very interesting that the increased level of SUA was related to ICRI only in women. Generally speaking, there has been no difference in the association between SUA and cardiovascular risk in men and women [1-3], but our results showed a clear difference between genders. In this regard, a post hoc analysis of the LIFE trial indicated that the association between the level of SUA and cardiovascular outcomes was significant only in women after adjustment for the Framingham risk score [12]. Likewise, data from a population study revealed an association between SUA and silent brain infarcts in women, but not in men [13]. Moreover, SUA was shown to correlate to renal resistive index only in women [11], strengthening the assumption that this gender exhibits higher propensity to SUA-induced microvascular damage. A potential explanation for such gender differences is not clear, but may include variation in sexual hormone profile. Menopause is associated with increased SUA levels because of the uricosuric effect of estrogen [26,27], which might have influenced our results. However, the lack of impact of menopause status on our regression analysis seems to weaken this assumption. Therefore the exact underlying mechanisms should be further investigated.

SUA was not related to carotid elasticity/stiffness indexes and IMT in our sample. These findings agree with previous studies showing no relationship between SUA and the structure of large arteries in hypertensive subjects [7,8], but are in contrast to data obtained in other hypertensive populations [4-6]. The reasons for such discrepancies are not apparent, but it is possible that differences in clinical and ethnical features among the studied populations played a role in this regard. Furthermore, in many studies, aortic stiffness, instead of carotid elastic properties, was studied [4,5]. In this regard, it is possible that there might be differences in structural and functional features between these two arteries [28]. On the other hand, we believe that the lack of relationship was also explained by the high prevalence of metabolic syndrome in our sample, since SUA may not be related to carotid structure among subjects with this phenotype [29,30].

A potential limitation of our study was that its cross-sectional design may limit our ability to infer a causal relationship between SUA and ICRI. Furthermore, it must be acknowledged that the majority of patients were using antihypertensive medications, which might be a confounding factor in the analysis. In this regard, thiazide and loop diuretics are known to increase SUA levels [31], while the angiotensin receptor blocker losartan has unique uricosuric properties [32]. In our sample, the use of thiazide or loop diuretics was observed in 76% of women and 84% of men, while losartan had been used by 14% of women and 7% of men. Nevertheless, results of univariate analysis showed no significant correlation between these aforementioned antihypertensive medications and SUA levels in both genders (data not shown). We also found that antihypertensive medications exhibited no correlation with any studied carotid variable in both genders and forced inclusion of antihypertensive medications in the regression models did not change the association between SUA and ICRI in hypertensive women.

## Conclusions

The results of the present study demonstrated a previous unknown association between SUA and ICRI solely in hypertensive women. Further studies are warranted to evaluate whether this association contributes to explain uric acid-related adverse cardiovascular prognosis in hypertensive subjects of this gender.

## Abbreviations

SUA, Serum uric acid; IMT, Intima-media thickness; ICRI, Internal carotid artery resistive index.

## Competing interests

The authors declare that they have no competing interests.

## Authors’ contributions

JAAC, JRMS and WNJ designed research; JAAC, JRMS, MCFS, RPM, VRB and JAPM conducted research and collected data; JAAC, JRMS and WNJ analyzed and interpreted data; JAAC and WNJ wrote paper; All authors read and approved the final manuscript.

## Pre-publication history

The pre-publication history for this paper can be accessed here:

http://www.biomedcentral.com/1471-2261/12/52/prepub
